# Reactive Cobalt–Oxo Complexes of Tetrapyrrolic Macrocycles and *N*-based Ligand in Oxidative Transformation Reactions

**DOI:** 10.3390/molecules24010078

**Published:** 2018-12-26

**Authors:** Atif Ali, Waseem Akram, Hai-Yang Liu

**Affiliations:** Department of Chemistry, The Key Laboratory of Fuel Cell Technology of Guangdong Province, South China University of Technology, Guangzhou 510641, China; atifali.chemistry@gmail.com (A.A.); waseem66@163.com (W.A.)

**Keywords:** cobalt–oxo complex, tetrapyrrolic macrocycles, *N*-based ligand, identification

## Abstract

High-valent cobalt–oxo complexes are reactive transient intermediates in a number of oxidative transformation processes e.g., water oxidation and oxygen atom transfer reactions. Studies of cobalt–oxo complexes are very important for understanding the mechanism of the oxygen evolution center in natural photosynthesis, and helpful to replicate enzyme catalysis in artificial systems. This review summarizes the development of identification of high-valent cobalt–oxo species of tetrapyrrolic macrocycles and *N*-based ligands in oxidation of organic substrates, water oxidation reaction and in the preparation of cobalt–oxo complexes.

## 1. Introduction

**Figure 1:**
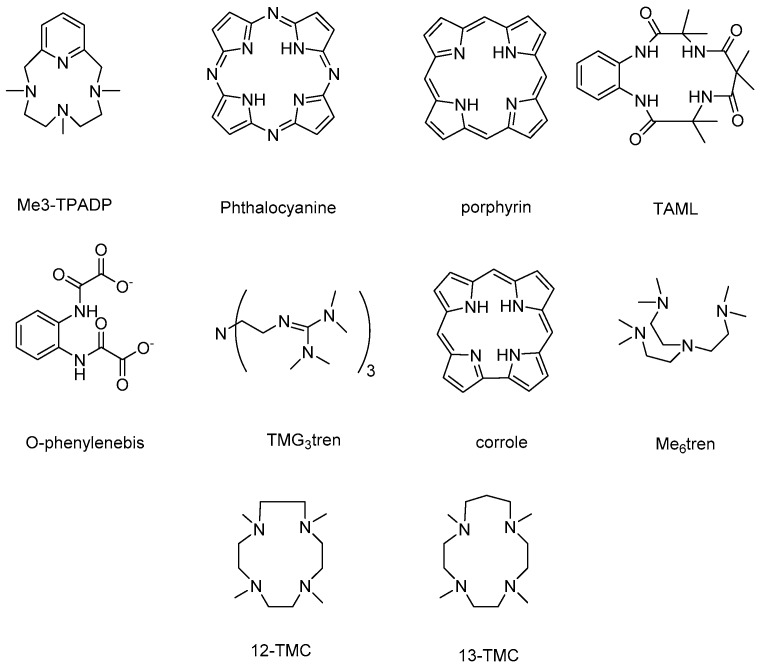
Some of the most used tetrapyrrolic macrocycles and *N*-based ligands used to stabilize high-valent cobalt–oxo complexes.

In biological systems, metalloenzymes, typically containing Mn, Fe and Cu centers, are known to catalyze a wide range of reactions including aliphatic and aromatic C–H hydroxylation, epoxidation, desaturation, and heteroatom (S, N or O) dealkylation or oxidation [1,2]. It is well known that iron‒oxo species are the reactive oxidants in the catalytic cycle of heme [3] and non-heme iron enzymes [4]. Similarly, manganese–oxo complex has been suggested the key intermediate in oxygen-evolving center of photo-system II (PSII) [5,6,7]. The transition metal–oxo complexes of iron and manganese involved in artificial oxygen transfer and C–H bond activations reactions have been extensively reviewed [8,9,10,11,12,13]. Except for the early transition metal–oxo complexes, high-valent metal–oxo complexes of late transition metals, particularly cobalt–oxo complexes, are also highly reactive transient intermediates in cobalt-catalyzed C‒H bond activation and O‒O bond formation reactions [14,15,16], and they are considered to be more reactive then related iron‒oxo species due to a weak metal–oxygen bond [17,18]. Currently, clean energy production by maneuvering natural photosynthesis in water oxidation reactions to develop artificial photosynthesis [19,20,21] for efficient water splitting is a hot topic of research [22,23,24]. In particular, the cobalt oxides are often used materials for water oxidation to generate molecular oxygen [25,26,27,28]. The high-valent cobalt–oxo complexes of *N*-based ligand can be implicated as reactive species in the O–O bond-forming event during water oxidation [29,30]. Furthermore, cobalt complexes based on tetrapyrrolic macrocycles are often used in mimicking the peroxidase-like activity for the selective oxidation of organic substrates via high-valent cobalt(IV)–oxo intermediates [31,32]. Obviously, in the study of the reactive oxidants in these catalytic reactions it is essential to provide insight into their mechanism of reaction, allowing us to probe the critical step in these challenging reactions. However, the isolation and identification of these transient intermediates is considerable challenge. The cobalt–oxo complexes are not stable because cobalt has large number of d-electrons which produces strong electronic repulsion between the rich electron oxygen and cobalt center. Also, the strong oxidative environment will cause oxidative degradation of the ligand, making the high-valent cobalt–oxo complexes unstable. To reduce the electronic repulsion between cobalt and oxygen, and to avoid oxidative degradation, tetrapyrrolic macrocylces and *N*-based ligands with a different electronic environment were implemented to increase the stability of cobalt–oxo complexes (Figure 1). Mostly, these can only be characterized in situ by electron paramagnetic resonance (EPR) [33], X-ray absorption [34] and time-resolved Fourier-transform infrared (FT-IR) [16] spectroscopic methods. Computational studies were also carried out to understand the nature of species involved in water oxidation [35]. Recently, the isolation and/or identification of high-valent cobalt–oxo complexes has become a key topic in order to develop and understand the mechanism of artificial photosynthesis and to replicate enzymatic process in artificial reactions.

This review comprehends the high-valent cobalt–oxo complexes of tetrapyrrolic macrocycles and *N*-based ligands reported to date, along with outlooks in this intriguing research area. It has been divided into three sections: identification of cobalt–oxo species involved in oxidation of organic substrates; identification of cobalt–oxo species involved in heterogeneous and homogeneous water oxidation reactions; and preparation of high-valent cobalt–oxo complexes.

## 2. Cobalt–Oxo Species Involved in Oxidation of Organic Substrates

Cobalt–oxo species are involved in many of oxidative and C‒H bond activation reactions. The ligands used to generate cobalt–oxo species play a key role in stabilizing cobalt–oxo species. Also, to mimic the enzymes-like environment, different types of support are used as protein backbone for example cellulosic fiber and multiwall carbon nanotubes. These supports cannot alter the reaction mechanism however, precisely control the generation of reactive intermediate, which also determines the activity, durability and stability of the complexes [36,37,38].

**Scheme 1:**
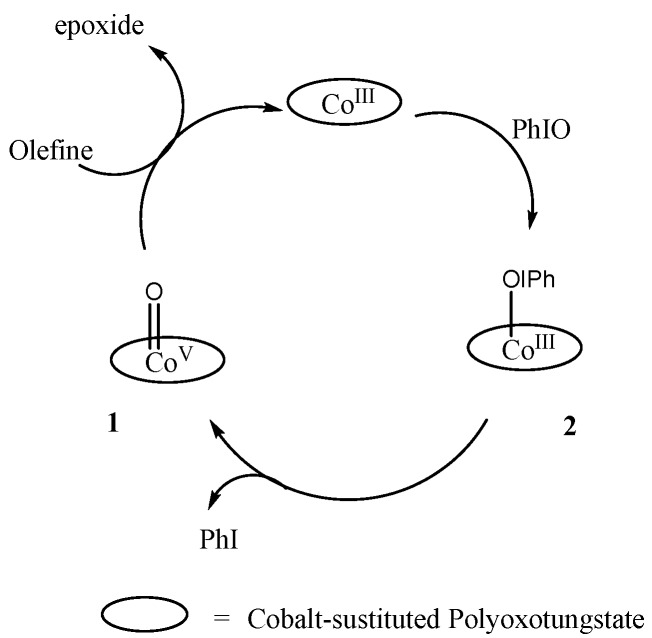
Proposed mechanism for oxidation of alkene by cobalt polyoxotungstate using PhIO as oxidant [39].

Nam et al. reported [39] the catalytic oxidation of alkene and alkane using cobalt-substituted polyoxotungstate and employed different oxidants such as iodosylbenzene, potassium monopersulfate and *m*-CPBA. Cobalt-substituted polyoxotungstate was proved to be a good catalyst. They proposed the involvement of different cobalt–oxo species with the different oxidants. Two possible species may form with iodosylbenzene, high-valent cobalt(V)–oxo 1 and cobalt–iodosylbenzene adduct 2 (Scheme 1). They suggested that complex 2 is responsible for oxygen transfer because cobalt cannot be obtained in +5 oxidation state. KHSO_5_ and *m*-CPBA predict the involvement of cobalt(III)–oxygen adducts as oxygen transfer complex. Isotopically labeled water (H_2_^18^O) is a useful experimental tool to investigate the involvement of high-valent cobalt–oxo species in cobalt-mediated oxygen atom transfer reactions, but all the attempts to obtain ^18^O-labeled products have failed. Furthermore, porphyrins are extensively used to get stable metal–oxo complexes [13]. Therefore, porphyrins with a different electronic environment were used to stabilize cobalt–oxo species [40,41]. Cobalt(IV)‒oxo [40] and cobalt(IV)–oxo porphyrin radical [41] were proposed to be involved in C‒H bond activation reaction. These species are quite reactive towards the oxidation of alkane and alcohol, respectively. However, there is no experimental evidence to support presence of cobalt–oxo species due to instability. Likewise, a cobalt(IV)–oxo species was reported [42], based on the tetraanionic cobalt(II) complex of (^Br^HBA-Et)H_4_, *N*,*N*′-(ethane-1,2-diyl)bis(5-bromo-2-hydroxybenzamide), that provides a strong ligand field. Consequently, this specie was stable enough to be characterized by EPR and ESI-MS spectroscopy analysis. Also, the presence of high-valent cobalt(IV)–oxo porphyrin was reported during the oxidation of alcohol to benzaldehyde by molecular oxygen in the presence of isobutyraldehyde, using bifunctional hybride catalyst originated from cobalt tetra(4-sulfonatophenyl)porphyrinate anion [43] and a cationic *meso*-tetrakis (1-methyl-4-pyridyl) cobalt porphyrin immobilized in montmorillonite interlayers [44]. The presence of a cobalt(IV)–oxo specie was predicted by an ^18^O-labeled experiment of product [43]. The turnover frequency and catalytic yield was higher in the prior case. Later, the cobalt(IV)–oxo porphyrin was generated [45] by the oxidation of cobalt porphyrin Co(TPFPP)(CF_3_SO_3_) utilizing *m*-CPBA as oxidant in solvent mixture of CH_3_CN and CH_2_Cl_2_. Incorporation of H_2_^18^O in the catalytic oxidation demonstrated the presence of ^18^O-labeled alcohol in the product, which is evidence for the presence of cobalt–oxo species. Furthermore, cobalt(V)=O and cobalt(IV)=O were generated [46] by the oxidation of a mononuclear non haem cobalt(III) [Co((bpc)Cl_2_][Et_4_N] (H_2_bpc=4,5-dichloro-1,2-bis(2-pyridine-2-carboxamido)benzene) complex of a tetradentate ligand containing two deprotonated amide moieties with PhIO. Oxidation of the cobalt(III) complex generated cobalt acylperoxo intermediate, which on the heterolytic and homolytic cleavage of O‒O bond generated respective cobalt(V)–oxo and cobalt(IV)–oxo species. These species are also not enough stable to be characterized spectroscopically. Similarly, a cobalt(IV)=O specie based on isoindole-core ligand was proposed [47] as a reactive intermediate, during the stereoselective oxidation of alkane using *m*-CPBA as oxidant. The kinetic isotopic effect and ^18^O-labeled experiment predict the involvement of cobalt–oxo species. Recently, the involvement of a high-valent cobalt(IV)=O radical cation was proposed [48] during the reduction of O_2_. The dianionic pentadentate ligand system based on bis-pyrazolyl diaryl borate arms attached to a 2,6-substituted pyridyl frame was used to stabilize the cobalt(IV)–oxo intermediate. Cobalt(IV)=O radical cation was generated by the cleavage of Co‒O bond, and examined theoretically and experimentally. A density functional theory (DFT) calculation suggests the presence of maximum electron density at oxygen 70% with Co‒O bond length of 1.67 Å.

**Figure 2:**
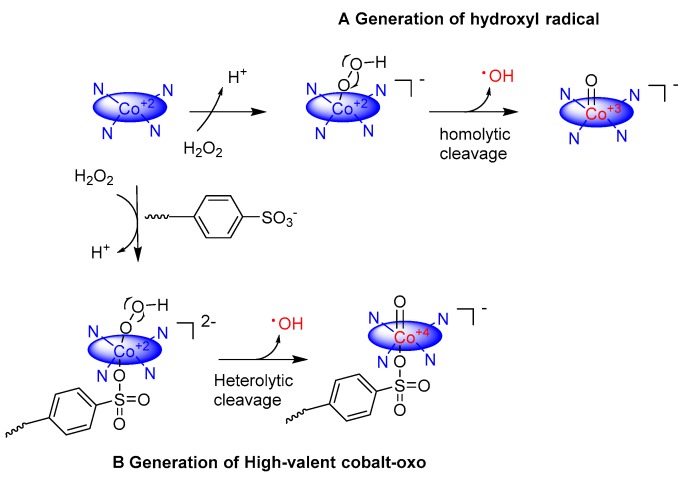
Possible pathway for the formation of active species in cellulosic fiber-bonded cobalt phthalocyanine (CoPc) H_2_O_2_ system. (**A**) Generation of hydroxyl radicals without ligand dodecylbenzenesulfonate (LAS) by the homolytic cleavage of the peroxide O‒O bond; (**B**) Generation of cobalt–oxo with LAS by the heterolytic cleavage of the peroxide O‒O bond [54].

Moreover, to mimic the enzyme activity for controllable catalytic oxidation, researchers made extensive efforts to develop and discover functional materials having properties intrinsic to enzymes. Many transition-metal complexes were prepared [49,50,51] to mimic the expected features of enzymes, such as selectivity and steric accessibility, but these do not present the said features due to the non-natural environment. A catalyst which is a replication of enzyme should possess a suitable cavity or cleft for accessibility of substrates and introduction of functional groups that act as active sites within the cavity [52,53]. Enzymatically inspired catalytic system was prepared by using cobalt tetraaminophthalocyanine (CoTAPc) as a catalyst supported by ordered-mesoporous-carbon (OMC) for controllable activation of hydrogen oxide (H_2_O_2_) to generate stable cobalt–oxo intermediate [32]. Ordered-mesoporous-carbon provides the steric environment for a substrate to attach with active sites and protects the active sites against the external interface. However, a disadvantage of hydrogen peroxide is the formation of hydroxyl radical that is highly reactive, so it decreases the selectivity. A fifth ligand dodecylbenzenesulfonate (LAS) is employed to inhibit the production of hydroxyl radical. This fifth ligand also helps to generate high-valent cobalt(IV)–oxo specie by heterolytic cleavage of peroxide O‒O bond. The involvement of cobalt–oxo specie was corroborated by the results of semiempirical quantum-chemical PM6 calculations. Similarly, a modification in the tetrapyrrolic macrocycle of cobalt tetraaminophthalocyanine (CoTAPc) was made by the attachment of epoxy compound 2,3-epoxypropyl triethylammonium chloride (EPTAC), to obtain a new catalyst with positively charged quaternary ammonium salt chain (OMC-CoTAPc-EPTAC) [31]. The modified catalyst displays high catalytic activity especially for negatively charged substrates. The free radical trapping EPR analysis using 5,5-Dimethyl-1-pyrroline *N*-oxide (DMPO) as a free radical scavenger did not detect DMPO-˙OH and DMPO-˙OOH signal, ruling out the free radical type mechanism. That is why, the cobalt(IV)–oxo complex was proposed as a reactive intermediate due to the heterolytic cleavage of O‒O bond of peroxide. Moreover, cellulosic fiber could play the role of the protein backbone in enzymes, providing an enzyme-like environment with enhanced regioselectivity to remove organic dyes and improve the stability of intermediate generated. A catalyst was developed based on cellulosic fiber-bonded cobalt phthalocyanine catalytic entity to activate hydrogen peroxide in order to generate cobalt–oxo specie [54]. The reaction channel was controlled by linear alkylbenzene sulfonate (LAS). High-valent cobalt(IV)–oxo specie **4** was generated by the heterolytic cleavage of peroxide O‒O bond and homolytic cleavage generate cobalt(III)–oxo specie **3** (Figure 2).

**Figure 3:**
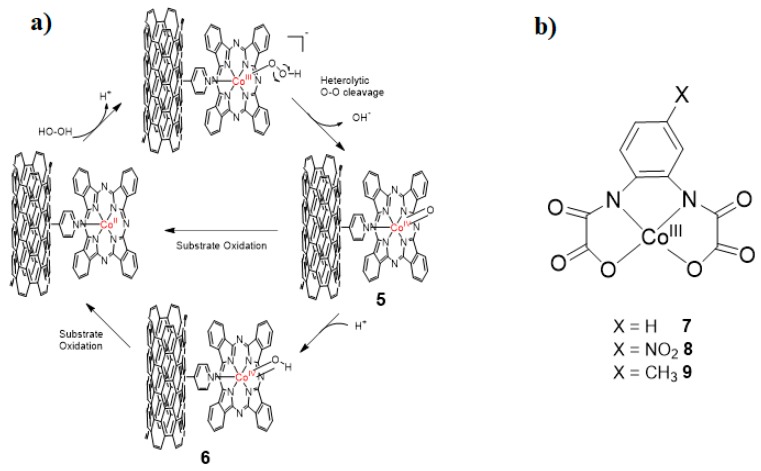
(**a**) Proposed mechanism of activation of H_2_O_2_ catalyzed by CoPc-PyMWCNTs for oxidation of substrates at pH = 10 [59]. (**b**) structures of cobalt(III) complexes involved in the activation hydrogen peroxide [62].

**Scheme 2:**
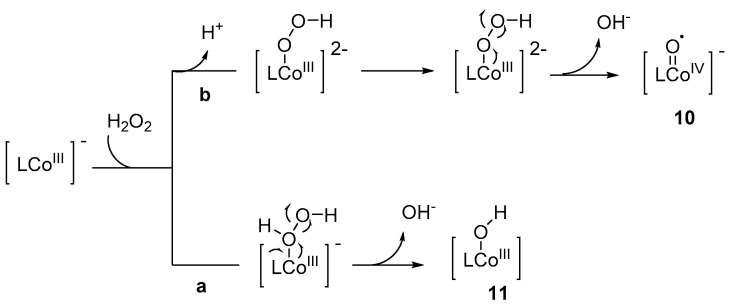
Proposed mechanism for the formation of cobalt–oxo species (L = bis-benzoamido) [60].

The in situ X-band EPR analysis was conducted at room temperature in the presence of LAS demonstrating a signal at g_eff_ = 2.099 identifying the presence of Co^IV^ with spin state (S = ½). Usually, metal–oxo species have been detected at low temperature [55,56]; a high oxidation state of PcCo^IV^=O was observed at room temperature, presenting high stability of complex, auto-oxidation protected by cellulose matrix. Cellulosic fiber bonded cobalt phthalocyanine (CoPc) can also activate peroxymonosulfate [57]. The oxidation activity of catalyst is remarkably enhanced in the presence of bicarbonate ion (HCO_3_^−^) due to the generation of (PcCo^IV^=O) by the heterolytic cleavage of O–O bond of peroxymonosulfate. Later, the same group [58] employed the multiwall carbon nanotubes (MWCNTs) as protein-like backbone anchored on cobalt phthalocyanine (CoPc) for peroxidase like activation of hydrogen peroxide. The anchoring of catalytic entity on MWCNTs decreases the diffusional mass transfer process (DMTP) and enhances the resistance of CoPc-MWCNTs oxidative decay. The introduction of linear alkylbenzene sulfonates (LAS) facilitates the heterolytic cleavage of O‒O bond of peroxide to generate cobalt(IV)–oxo species. Furthermore, pyridine functionalized MWCNTs were produced and axially coordinated on the cobalt phthalocyanine (CoPc), generating a catalyst with increased catalytic activity and stability [59]. The heterolytic cleavage of O‒O bond of hydrogen peroxide to produce cobalt(IV)–oxo without presence of any fifth ligand. The high-valent cobalt(IV)–oxo was analyzed by in situ ESI-MS and density functional theory. The DFT calculated bond length of Co‒O bond is 1.806 Å and unpaired electron spin populations are mainly on the oxygen. Cobalt(IV)–oxo **5** was generated at pH = 10, the catalytic cycle starts with the coordination of OOH^−^ with Co^II^, and the heterolytic cleavage of O‒O occurs with the release of OH^−^ (Figure 3a). Moreover, non-haem cobalt(III) oxamate anion could also be used to stabilize high-valent cobalt(IV)–oxo species [60]. The oxidation of industrial contaminants was reported [61] by immobilizing non haem cobalt(III) complex [Co^III^(opbaX)]^−^(opbaX = 4-X-o-phenylenebis(oxamate) on pyridine-modified MWCNTs, where pyridine acts as a fifth ligand. Similarly, cobalt(III) complexes of [Co^III^(opbaX)]^‒^(opbaX = 4-X-o-phenylenebis(oxamate), X = H, NO_2_, CH_3_) with different substituents was reported [62] for accelerating heterolytic cleavage of hydrogen peroxide to imitate the essential and general principle of natural enzymes without using any fifth ligand (Figure 3b). An ESI-MS and EPR trapping technique revealed the presence of cobalt(IV)–oxo reactive specie. The generation of cobalt–oxo species depends on the electronic environment of substituent. In Scheme 2 pathway (b) the electron rich cobalt complex favors the homolytical cleavage of the hydroperoxide O‒O bond while the electron deficiency favors the heterolytic cleavage with generation of (Co^IV^=O)**10**. The tendency of generation of (Co^IV^=O) was in order **8** > **7** > **9**. Density functional theory also demonstrated that electron withdrawing group helps in pulling electron and lowering the corresponding energy levels. Keeping in mind the concept of the “oxo wall” [63], another pathway (a) also proposed, heterolytic cleavage of O‒O generate the ligand based radical intermediate OH–Co^III^(opbaX)**11**, in which ligand transfers one electron to cobalt and cobalt transfers it to oxygen.

Our group recently [64] reported the catalytic oxidation of alkene using four cobalt(III) corroles of different electronic environment F_0_C-Co, F_5_C-Co, F_10_C-Co and F_15_C-Co employing various oxidants. The in situ ESI HR-MS analysis of styrene oxidation with KHSO_5_ predicts the presence of high-valent cobalt(V)–oxo complex as active intermediate. The in situ X-band CW EPR analysis revealed a signal at g = 2.0135 for the presence of cobalt–oxo specie.

## 3. Cobalt–Oxo Species Involved in Water Oxidation Reaction

Water oxidation is a process that involved the four-electron-four-proton oxidation of water to evolve O_2._ In natural photosynthesis, sunlight is converted to chemical energy by the oxidation of water [20]. As a consequence, understanding nature’s water oxidation mechanism in photosystem II has been the focus of research for the development of artificial water oxidation catalyst. The development of efficient water oxidation catalysts with minimal cost is a challenge [65,66,67,68]. Various water oxidation catalysts were developed to understand the O‒O bond formation event in natural photosynthesis to evolve oxygen. Cobalt is the most abundant and cheap earth metal. Cobalt oxide materials are among the most promising catalyst for water oxidation [69,70,71] and cobalt–oxo species are involved in the O‒O forming event of water oxidation.

**Scheme 3:**
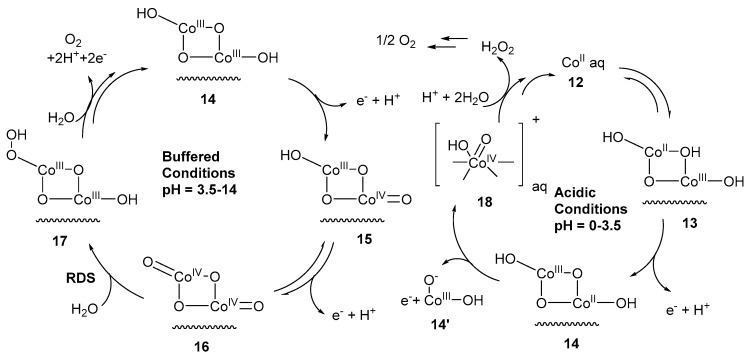
Mechanism for oxidation of water by cobalt oxide under acidic and basic conditions [74].

Frei et al. reported [16] the photocatalytic water oxidation using cobalt oxide. The water oxidation was carried out in the presence of photosensitizer [Ru(bpy)_3_]^2+^ (bpy = 2,2′-bipyridine) that creates a hole and S_2_SO_8_^−2^ as an electron acceptor. The FT–IR characterization revealed the involvement of two intermediates with absorption band at 1013 cm^−1^ and 840 cm^−1^. The band at 1013 cm^−1^ was assigned to Co(III)OO (fast site) group with a neighboring hydroxyl group. Incorporation of H_2_^18^O shifts the peaks at 995 cm^−1^ and 966 cm^−1^. The shifting of frequency agrees well with the presence of superoxide moiety on a metal-oxide surface [72,73]. The superoxide surface intermediate causes the three-electron water oxidation. The band at 840 cm^−1^ was assigned to Co^IV^=O (slow site) surface species. No change in the spectrum was observed by the incorporation of H_2_^18^O, ruling out the presence of any peroxide intermediate. From the mechanistic point of view, Co^IV^=O was generated by the oxidation of surface group Co(III)–OH. At the fast site catalytic species, catalytic turn over frequency is at least 10 times more than slow site catalytic species, because it has no neighbor hydroxyl group. Furthermore, Stahl et al. reported [74] the water oxidation employing cobalt oxide as an electrocatalyst, and proposed the involvement of (Co^IV^‒O) as reactive specie. The EPR analysis with signals at g-values 2.59, 2.17 and 1.99 revealed the presence of multiple paramagnetic species during water oxidation, possibly arising from (Co^IV^‒O) sites in the catalyst with a different coordination environment. The mechanism of water oxidation is pH dependent, at acidic pH homogeneous catalysis leading to H_2_O_2_ production, while at pH above 3.5 heterogeneous catalysis takes place, generating O_2_ from four-electron water oxidation (Scheme 3). The oxidation of **12** produced **14** (**12**→**13**→**14**) corresponds to a 3H^+^/e^−^ process. Subsequently, 1e^−^ oxidation generated specie **15** corresponding to 7H^+^/3e^−^ process. Further, oxidation of **15** afforded **16**. A key step to evolve oxygen is the nucleophilic attack of water at **16** to produce **17** [75,76,77]. Under the acidic pH, the PCET-mediated formation of **11** was prevented (Scheme 3). The oxidation of **10** produced **18** that dissolve from surface. The intermediate specie **18** invoked the homogeneous oxidation of water to H_2_O_2_ [78]. Similarly, bridging cobalt(IV)–oxo [79] and terminal cobalt(IV)–oxo radical [80] species were proposed as reactive catalytic sites for water oxidation, employing amorphous cobalt oxide. X-ray absorption near the edge provides the insight that the generation of high-valent (Co^IV^‒O) depended on the potential applied and pH. The edge position of the spectra was taken at pH = 7 and pH = 9 differs by about 1.0 eV by keeping potential constant at 0.95 V, and edge position of the spectra were taken at pH = 7 by increasing electrode potential from 0.95 to 1.34 V differs by about 1.2 eV [79]. However, the study of cobalt–oxo species involved in water oxidation was difficult because in oxygen evolving catalysis (OEC), large number of spectroscopically active backgrounds species are present which limits their detection and characterization.

**Scheme 4:**
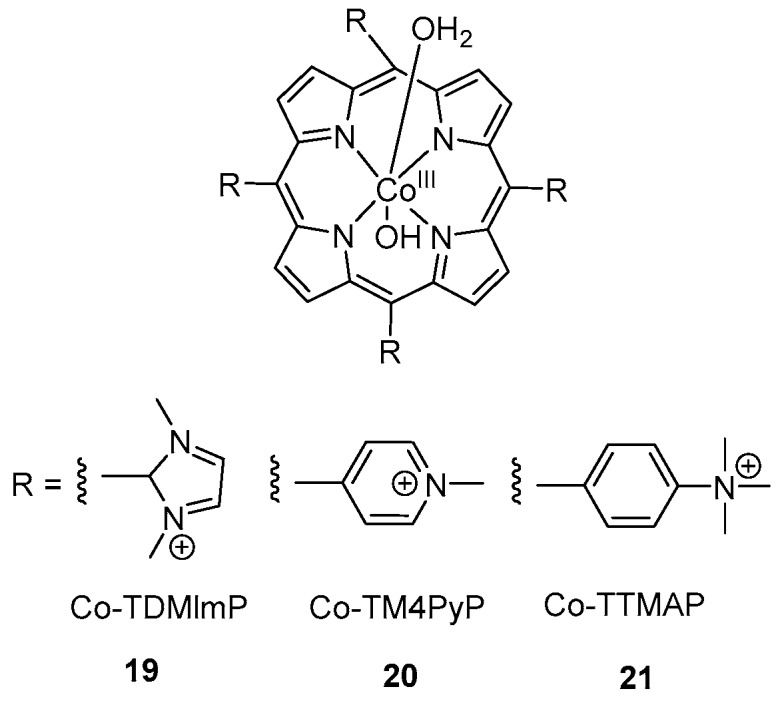
Molecular structures of cobalt porphyrins employed as water oxidation catalyst.

**Scheme 5:**
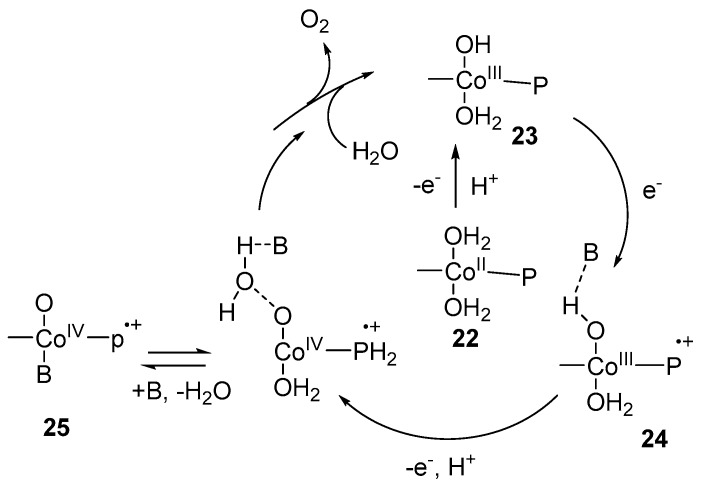
Proposed mechanism of water oxidation catalyzed by cobalt porphyrins [92].

**Scheme 6:**
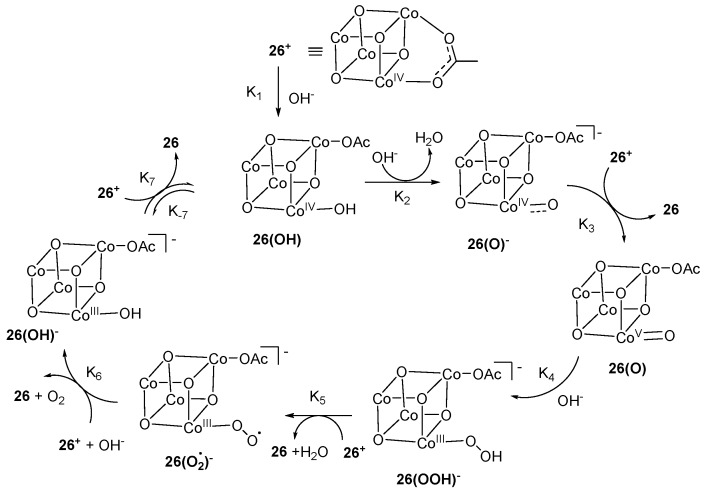
Generation of high-valent Co^V^=O **26(O)** during water oxidation by molecular cobalt cubane Co_4_O_4_(OAc)_4_py_4_ [96].

Consequently, suitable catalysts to reduce background active species are needed to design. *N*-based ligands have attractive properties to be used as homogenous molecular water oxidation catalysts [81]. Recently, a significant number of catalysts are developed based on single site and multinuclear transition metal including Mn, Fe, Co, Cu, Ru and Ir [82,83,84,85,86,87]. The biggest challenge is to find a suitable coordination environment because the metal–ligand bond opposite a metal–oxygen bond can be compromised at higher redox level leading the catalyst to be susceptible to degradation [88]. So, single site *N*-based ligand homogeneous catalysts of cobalt were developed utilizing stable pentadentate ligand environment of 2,6-(bis(bis-2-pyridyl)methoxy-methane)-pyridine [89] and 6-(bis(bis-2-pyridyl)-methoxymethane)pyridine [30] for water oxidation. The electrochemical studies revealed that over pH range 7.6–10.3 an oxidation event was observed at +1.43 V vs. NHE corresponding to [Co^IV^–OH]^3+^/[Co^III^–OH]^2+^ with significant rise in current. This signal is not classified as PCET because *E*_1/2_ is static over this pH range. A pH dependent step was observed at pH > 10.3 corresponding to [Co^IV^=O]^2+^/[Co^III^–OH]^2+^ which is consistent with PCET. High-valent [Co^IV^=O]^2+^ species evolves O_2_ by the nucleophilic attack of H_2_O [89,90]. An alternative pathway proposed that the attack of OH^-^ at [Co^IV^–OH]^3+^ in the rate determining step will evolve O_2_ [30]. Likewise, [Co^IV^‒O] specie was proposed as reactive intermediate during water oxidation at basic pH using cobalt-porphyrins as catalyst [91]. Similarly, Groves and Wang reported [92] the single site homogeneous water oxidation catalyst, employing a series of cobalt porphyrins **19**, **20** and **21** (Scheme 4). A high-valent Co^IV^-porphyrin cation radical acts as reactive intermediate. The electrochemical experiment provides the evidence for the formation of high-valent Co^IV^–O specie. The redox event at 250 mV vs. Ag/Cl reference represents the resting state of catalyst H_2_O–Co^III^–OH **23**. The observed anodic features at ~1 V demonstrates the oxidation of Co^III^ porphyrin to Co^III^ porphyrin radical cation (+P-Co^III^–OH) **24**. As first oxidation occurred before the onset potential of WOC catalytic current, so +P–Co^III^–OH is not the reactive oxidant in this system. The second oxidation at 1320 mV generates a reactive high-valent Co^IV^–O porphyrin radical cation **25**. The key step for O‒O bond formation is the nucleophilic addition of H_2_O to +P–Co^IV^–O **25** to form Co-hydroperoxo or peroxo which further oxidized to evolve O_2_ as shown in Scheme 5. Likewise, photo-induced generation of Co^IV^=O as active oxidant for the water oxidation was reported [93] based on a cobalt(II) complex of salophen ligand. Moreover, a high-valent Co^IV^O complex isoelectronic to Co^V^O was reported [29] to act as active specie to generate O_2_ based on a cobalt(III) complex of *N*-based ligand bTAML (bTAML = biuret-modified tetraamidomacrocyclic) ligands. The complex [Co(O)(bTAML)]^1−^ cannot be characterized by spectroscopic techniques due to the non-innocent nature of the ligand except UV-vis spectra. The same specie was generated by the one electron oxidation using cerium ammonium nitrate in the presence of ZnCl_2_. The HR-MS analysis revealed the *m/z* = 497.026 corresponding to [Co^IV^(O)(Zn)(bTAML)(H^+^)]. Further, Nocera et al. reported [94] the dicobalt oxidized site Co(III)_2_Co(IV)_2_ during water oxidation using cobalt cubane modified by pyridine ligands that can stabilize tetracobalt core. This pyridine-modified cobalt cubane has molecular nature and termed as molecular cubane. Electrochemical investigation demonstrated two reversible oxidation events at *E*_0_(1) = 0.3 V and *E*_0_(2) = 1.25 V corresponding to Co(III)_3_(IV)/Co(III)_4_ and Co(III)_2_(IV)_2_/Co(III)_3_(IV). X-ray absorption spectroscopy also confirms the presence of Co(III)_2_(IV)_2_ specie. The adjacent terminal Co^IV^=O species in cubane provide a site for direct O–O bond formation by radical coupling to evolve O_2_. Likewise, the proton-coupled electron transfer generation of (Co^IV^‒O) was also reported [95] using molecular model cubane, [Co_4_O_4_(CO_2_Me_2_)_2_(bpy)_4_]. Furthermore, molecular cobalt cubane Co_4_O_4_(OAc)_4_py_4_ **26** [96] and a series of modified molecular cobalt cubane with electron rich and electron poor groups [97] were reported to understand the nature of high-valent cobalt–oxo species involved in the water oxidation reaction. The electrochemical studies of **26** revealed the presence of only one fully redox couple from pH 4 to pH 10 at *E*_1/2_ = 1.25 V corresponding to Co(III)_4_/Co(III)_3_(IV) redox. The increase of pH to 12 produced a significant anode wave current and bubble formation, consistent with the oxidation of hydroxide to oxygen. No change in the current intensity was observed in the presence of EDTA, ruling out the possibility of heterogeneous water oxidation due to the presence of Co^II^ oxide. The ESI-MS analysis by incorporating 97% enriched Na^18^OH observed the presence of 90% ^36^O_2_. No evidence for the exchange of ^18^O-oxygen between **26** revealing that only terminal oxo/hydroxide specie was involved in O‒O bond formation. The reaction of protonated **26^+^** and hydroxide showed the importance of the cobalt(IV) oxidation state in O_2_ formation. The generation of cobalt(V)=O **26(O)** was proposed by PCET before the evaluation of O_2_ as shown in Scheme 6 [96]. The protonated **26^+^** reacts with hydroxide ion to produce **26(O)^−^** which further oxidized to cobalt(V)=O **26(O)**. The specie **26(O)** had acted as reactive intermediate to evolve O_2_. Involvement of high-valent cobalt(V)=O complex during water oxidation was also theoretically proposed [98,99,100,101]. Corroles are analogous of porphyrin which have one carbon less than porphyrin and can stabilize metals in a higher oxidation state. A high-valent Co^V^=O specie suggested [102] to act as reactive specie during water oxidation by using series of cobalt corroles with different axial ligand. Electrochemical study of cobalt corroles represent two reversible oxidation events at *E*_1/2_ = 0.75 and *E*_1/2_ = 1.32 V vs. NHE corresponding to Co^IV^/Co^III^ and Co^V^/Co^IV^ redox couples, respectively. Nucleophilic attack of the water at Co‒O bond to generate Co-hydroperoxo specie is the key step to evolve O_2_. Cobalt corroles with electron-donating ligands are more reactive because it causes the Co‒O bond to be weaker and nucleophilic attack become easier.

## 4. Preparation of Cobalt–Oxo Complexes

The isolated preparation of cobalt–oxo complexes have two major problems (1). Ligands used to stabilize cobalt–oxo complexes are prone to oxidation (2). Electronic repulsion forces between the d-electron of cobalt and electron of the oxygen. Chemists are focusing on how to overcome these problems to prepare cobalt–oxo complexes.

**Scheme 7:**
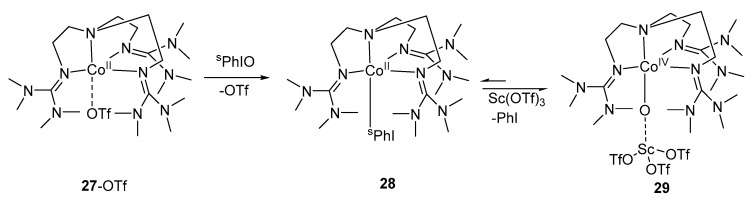
Preparation of high-valent {Co^IV^-O-Sc^3+^} complex **29**.

**Scheme 8:**
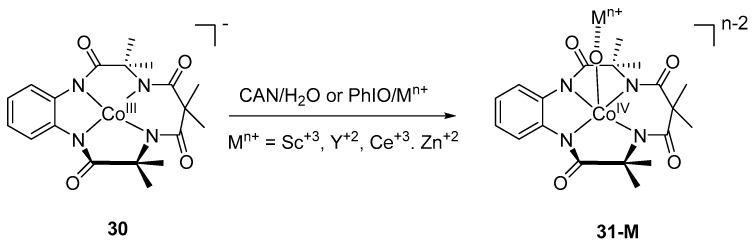
Oxidation of complex **30** to **31-M** by CAN/H_2_O or PhIO/M^n+.^

**Scheme 9:**
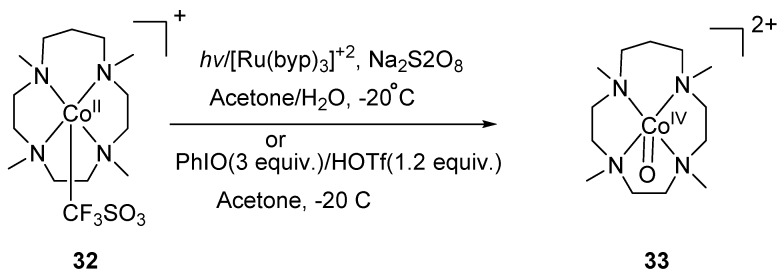
Preparation of a mononuclear non-haem cobalt(IV)–oxo complex [(13-TMC)Co^IV^(O)]^2+^.

Ray et al. reported [103] the first preparation and isolation of terminal cobalt(IV)–oxo complex using the *N*-based tetradentate tripodal ligand TMG_3_tren (tris[2-(*N*-tetramethylguanidyl)ethyl]amine). The {Co-O} unit was stabilized by the Lewis acid interaction with Sc^+3^ ion, generating {Co-O-Sc}^+3^ unit. The complex **29** was obtained by two electron oxidation of **27**-OTf in the presence of Sc(OTf)_3_ (Scheme 7). The complex **29** was characterized by electrospray mass spectrum, EPR and X-ray absorption spectroscopy, and was reactive towards oxidation of triphenylphosphine and dihydroanthracene. The same group two years later reported [104] the square pyramidal cobalt(IV)–oxo with enhanced stability based on the tetraamido macrocyclic ligand (TMAL). The electrochemical study of **30** gave a reversible oxidation peak at 1.00 V vs. a saturated colomel electrode. This reversible oxidation peak suggests that Co^IV^ state is thermally and kinetically accessible. The one electron oxidation of **30** in the presence of cerium ammonium nitrate (CAN) afforded a blue-colored complex **31**-Ce with a half-life of 20 min. This blue complex can also be obtained by the oxidation of **30** with PhIO in the presence other redox-inactive metals like Sc^+3^, Y^+3^ and Zn^+2^ (Scheme 8). The complex **31-M** was characterized by cold-spray ionization time-of-flight mass spectrometry (CSI-TOF MS), X-band EPR spectrum, and X-ray absorption spectroscopy. All attempts to obtain resonance Raman spectrum have failed. The **31-Sc** complex demonstrated high reactivity in the hydrogen abstraction reaction and oxygen atom transfer reactions. The first fully spectroscopically characterized high-valent Cobalt(IV)–oxo complex **33** was generated [105] by the two electron oxidation of a cobalt complex of 13-TMC (2 mM) **32** by PhIO (3 equiv.) following conventional method in the presence of triflic acid (CF_3_SO_3_H, HOTf; 1.2 equiv.) in acetone (Scheme 9). The transient complex had a half-life of 3 h and was characterized by CSI-TOF MS, EPR and X-ray absorption spectroscopy. Resonance Raman spectroscopy considered as authentic technique to confirm the presence of metal–oxo complex [106,107]. The resonance Raman spectrum of **33** showed a band at 770 cm^−1^ which shifts to 736 cm^−1^ upon ^18^O-labelling of **33**. Recently, preparation of Co^III^≡O complex was reported [108] by using tris-(imidazol-2-ylidene)borate ligand PhB(tBuIm)_3_^−^. This complex was characterized by infrared (IR) and X-ray diffraction (XRD) spectroscopy. The length of Co‒O bond determined by XRD was 1.68 Å. DFT calculations revealed two Co‒O π* interactions with highest lying d_xz_ and d_yz_ orbitals. These orbitals support the presence of two π-bonds. This complex was thermodynamically unstable with half-life of 8 h.

## 5. Summary and Outlook

High-valent cobalt–oxo complexes are implicated as key intermediates in many of the oxidative transformation reactions and the water oxidation process. Identification of cobalt–oxo species in water-splitting reactions have been extensively studied. However, the transient nature of cobalt–oxo complexes limits their characterizations to in situ EPR, XAS and mass spectroscopy. Although different strategies, such as using ligands with different electronic environments or MWCNT supports, have been adopted to stabilize cobalt–oxo complexes, until now only one example of Raman characterization for cobalt (IV)=O complex using 1,4,7,10-tetramethyl-1,4,7,10-tetraazacyclotridecane ligand has been available. The isolation and identification of high valent cobalt–oxo species remains a great challenge. The design of a suitable *N*-based ligand which can stabilize coordinated cobalt atom in high oxidation might be the key step for the preparation of higher valent cobalt–oxo complexes, which will allow the full characterization and “slow motion picture” study of the factors controlling its reactivity.

## Abbreviations

TPFPP	*M*eso-tetrakis(pentafluorophenyl)porphinato dianion
CoTAPc	cobalt tetraaminophthalocyanine
EPTAC	2,3-epoxypropyl triethylammonium chloride
Pc	Phthalocyanine
TAPc	Tetraaminophthalocyanine
MWNCTs	Multiwall carbon nanotubes
DMPO	5,5-Dimethyl-1-pyrroline *N*-oxide
F_0_C-Co	Co(III) complex of 5,10,15-triphenylcorrole
F_5_C-Co	Co(III) complex of 5,15-bis(phenyl)-10-(pentafluorophenyl)corrole
F_10_C-Co	Co(III) complex of 5,15-bis(pentafluorophenyl)-10-phenylcorrole
F_15_C-Co	Co(III) complex of 5,10,15-tris(pentafluorophenyl)corrole
TMG_3_tren	(tris[2-(*N*-tetramethylguanidyl)ethyl]amine)
^s^PhIO	2-(*tert*-butylsulfonyl)iodosylbenzene
TMAL	Tetraamido macrocyclic ligand
13-TMC	1,4,7,10-tetramethyl-1,4,7,10-tetraazacyclotridecane
Co-TDMImP	Co(III) complex of 5,10,15,20-tetrakis(1,3-dimethylimidazolium-2-yl)porphyrin
Co-TM4PyP	Co(III) complex of 5,10,15,20-tetrakis(*N*-methylpyridinium-4-yl)porphyrin
Co-TTMAP	Co(III) complex of 5,10,15,20-tetrakis(*N*,*N*,*N*-trimethylanilinium-4-yl)porphyrin
